# Moral structuring of children during the process of obtaining informed consent in clinical and research settings

**DOI:** 10.1186/s12910-020-00540-z

**Published:** 2020-11-25

**Authors:** Anderson Díaz-Pérez, Elkin Navarro Quiroz, Dilia Esther Aparicio Marenco

**Affiliations:** 1 Department of Social and Human Sciences, Simón Bolívar University, Barranquilla, Colombia; 2 Corporación Universitaria Rafael Núñez, Faculty Of Health sciences, Cartagena de Indias, Colombia; 3 Faculty of Basic and Biomedical Sciences, Simón Bolívar University, Carrera 59 No 59-65 Barranquilla, Colombia

**Keywords:** Autonomy, Paternalism, Doctor–patient relationship, Consent, Informed consent in minors, Capacity, Children, Understanding, Moral, Practices, Pediatrics, Clinical, Investigator, Biomedical

## Abstract

**Background:**

Informed consent is an important factor in a child’s moral structure from which different types of doctor–patient relationships arise. Children’s autonomy is currently under discussion in terms of their decent treatment, beyond what doctors and researchers perceive. To describe the influential practices that exist among clinicians and researchers toward children with chronic diseases during the process of obtaining informed consent.

**Methods:**

This was a cross-sectional, qualitative study via a subjective and interpretivist approach. The study was performed by conducting semi-structured interviews of 21 clinicians and researchers. Data analysis was performed using the SPSS version 21® and Atlas Ti version 7.0® programs.

**Results:**

The deliberative and paternalistic models were influential practices in the physician–patient relationship. In the deliberative model, the child is expected to have a moral awareness of their care. The paternalistic model determined that submission was a way of structuring the child because he or she is considered to be a subject of extreme care.

**Conclusions:**

The differentiated objectification [educational] process recognizes the internal and external elements of the child. Informed consent proved to be an appropriate means for strengthening moral and structuring the child.

## Background

Informed consent is the communication process wherein the child (boy, girl, or adolescent) is informed of aspects related to their illness or the importance of their participation in a study. In countries such as Colombia and Spain, clinicians and researchers (C&R) are legally required to inform and obtain a child’s consent unless they have been proven to be mentally incapable of providing it.

This investigation sought to identify the dynamics involved when requesting informed consent because it aims to understand the tensions that may occur between the researcher or clinician and the mature child with autoimmunity and their family, since it is the latter who directly provide their approval by means of the so-called informed consent, by substitution or by representation.

Studies such as the one proposed by Royo, Prado, and Maíllo (2012) indicate that in order for the physician or researcher to consider that the moral elements have been achieved in the minor, there must be an initiative to promote the development of their sense of utility, moral capacity, and sense of belonging to something [1]. It is important to analyze this from a specific sociocultural context, such as the Colombian context, from its representations in the physician or researcher’s perception or meanings regarding the minor’s moral development since several influential practices are produced. These are specifically physician–patient relationships that depend on the respect or violation of the minor’s rights within the framework of the principles of bioethics.

Investigations that describe the dilemmas faced by researchers and doctors associated with obtaining informed consent from mature minors, to which they are obliged by law, do not emphasize or report the (imaginary) meanings regarding the minor’s autonomy and responsibility.

Research in this field has shown that it has not been easy to clearly and precisely determine when humans begin to be autonomous subjects and legally responsible for their actions. In this aspect, authors such as Gutiérrez et al. state that consent is a process by which children are provided basic information about an investigation provided they demonstrate “… the ability to express preferences and understand the nature and purpose of the research techniques.” [2].

In this order of ideas, Freeman states that children’s rights are very important, and therefore, they must be treated equally and even as autonomous beings [3], with the child’s “best interests” in mind, which may be gained through the means of consent [4]. Thus, developing a child’s autonomy is as important as developing that of any other person, regardless of what stage of development they are in. Conversely, Sayoux and Frometa state that research related to children’s autonomy is limited to a historical and philosophical description of the need to consider the parents in decision-making, beginning with informed consent, wherein the child is a secondary subject who is not considered capable of decision-making [5]. The family plays an important role in the interaction of complex networks in the parent–child relationship, which is reflected in the child’s dependence and vulnerability and their decision-making capability [6].

However, studies such as those conducted by Manson (2015) and Tucker (2016) refer to the need to implement a transitional paternalism where it is not the child’s competence that is evoked but their flexibility, which leads to the normative power to morally structure the child to take responsibility for their decisions to an admissible extent. More specifically, C&R should prefer the fair methods, which give greater respect to the child’s decisions, rather than the symmetrical alternative of whether to consent [6, 7].

C&R should be aware that childhood is a stage in which the child develops a basic dependence on others [8]; therefore, transitional paternalism appeals to an individual’s obligations to help others self-govern [7, 9]. Likewise, López states that C&R should recognize minors as moral subjects only in their particular and unique evaluative aspects and very slightly from a normative and universal outset. In other words, they should recognize them as subjects with a universal right but not beyond the developmental stage that they have reached [8].

Elements of dominance emerge as influential practices from the way C&R perceive children’s autonomy or their moral development; these are manifested in the physician–patient relationship during the process of obtaining informed consent. Accordingly, Sánchez Vázquez proposed a classification system that illustrates the type of physician–patient power relationships that emerges from an applied ethics perspective. This could explain the dynamics of informed consent, including paternal-moral, normative-legal and differential type relationships [10]. Influential relationships respond to different logics when addressing the ethical problem surrounding the figure of the child; for example, the paternal-moral relationship conceives the child as only a “social being,” which is the object of care. The normative-legal relationship introduces the universal dimension brought about by the human rights phenomenon, in which the child is already treated as a dignified being with full rights. Finally, the differential relationship considers the child’s particular and unique perspective, highlighting the constant alternation of each child between autonomy and subjective vulnerability [10].

In summary, the different types of physician–patient relationships help us reflect on a child’s autonomy and formulate moral structuring guidelines (educative) for each type of patient. It should be mentioned that current guidelines do not clearly describe when to request a child’s consent for research, especially when they show a capacity for understanding [11]. Several studies describe the minimum requirements of health personnel when children are placed in their care. Their needs should be considered when they are needed to provide consent without coercion, and they need to be called by their name, among other things that define the axiological, teleological, and deontological elements [12, 13], which contributes to the child’s moral strengthening. Thus, autonomy interpreted as the child’s moral maturity could depend on how they assume their self-care practices and even recognize the importance of accepting their participation in an investigation [14]. These elements could lead to the C&R treating the child as a moral subject worthy of respect, with agency or self-government capacity [7, 9, 15], which is demonstrated by their self-care practices [13]. Conversely, according to an analysis of practices adapted to international standards for research in children, it also helps to examine the possible dilemmas and tensions raised regarding a child’s autonomous capacity, which are constantly evolving from a particular social and historical context [2, 5, 8, 13]. In Colombia, as in other Latin American countries, it is considered a moral duty to evaluate the autonomy of the minor within the informed consent process, regardless of their age, especially if they exceed 12 years, which have demonstrated degrees of communication and understanding with the ability to make limited decisions.

Informed consent merges influential practices of a deliberative or submissive type. Thus, as Powell and Buchanan expressed: “It is a complex construct where particular values and beliefs influence the way of conducting research’ and thus perceiving the ‘other’.” [16] Undoubtedly, it is imperative to understand that beyond the principles of beneficence, justice, and respect as normative elements, the sociocultural aspects of children and their families must be considered; otherwise, it may lead to an extreme paternalistic relationship [4, 7, 14].

Clearly, children, who are a vulnerable population, require representation by their legal guardians regardless of the C&R’s perception of the child’s moral development [8], which is neither under discussion nor is considered a problem or ethical dilemma. The problem lies in acknowledging the “other,” that is, the child as a moral subject, which confirms the guardian’s decision owing to the understanding and moral structuring that they have received from clinicians or researchers through an educative process to which the family substantially contributes [7, 17]. Consequently, informed consent does not end when the child leaves the practice or agrees to participate in an investigation; conversely, it is strengthened when the child practices self-care, establishing their best interest in improving or contributing to scientific knowledge above the needs characteristic of their age [11, 13, 18].

A limited number of studies have identified the practices of researchers and clinicians regarding children’s participation in research and their consent for treatment [12, 13, 18]. Most studies have only provided a description of the individual abilities of children and adolescents (minors) and have not mentioned anything about the meaning of autonomy and influential practices that emerge from C&R to recognize the child’s autonomy during the process of obtaining informed consent [7, 17]. However, they prove that the ethical challenges regarding biomedical research in children and adolescents (minors) are not simple when considering the moral capacity to make decisions, which could promote a relationship of extreme paternalism [19].

Opel et al. believe that the participation of children in biomedical research is justified because they contribute to improving treatments and diagnostic methods and are important for establishing guidelines for the control and management of pediatric diseases because such research cannot be conducted in adults [20]. In this sense, informed consent could be obtained more for administrative reasons than for reasons owing to moral duty [13]. López mentions that C&R should consider the evaluation of the child’s moral development based on the articles of the United Nations Children’s Fund, which address the participation of children in research [9, 20–22]. Article 12 states that “Children who can establish their own opinions should have the right to express them and have them taken into account.” Article 13 states that “Children are free to not participate and should not be pressured.” Therefore, “participation is a right, not an obligation.” [8] Ultimately, the child’s decision is a subjective fact for C&R, and therefore, it should be interpreted according to the framework of bioethical principles, in which the principle of beneficence prevails over the principle of autonomy without prior evaluation. This statement indicates that the conception of beneficence could be considered to be paternalistic [23, 24].

The child’s moral development should not be considered to be a limitation but a variation in the exercise of obtaining consent, while taking into account the child’s reasoning and common sense expressed in a language adapted to their cognitive development [13]. The child’s will to volunteer must be free but with the joint support of their guardian, C&R, family, and environment (heteronomy) [7, 10, 17, 25]. According to Kottow (2007), “the ethical concept of informed consent depends on the abolition of all unjustified medical paternalism.” [26] Therefore, the aim of this study was to describe the influential practices that exist between C&R and children with chronic diseases during the process of obtaining informed consent expressed in the physician–patient relationship.

## Method

A qualitative study was proposed to examine human behavior and the motives that govern it based on the social reality of a practice emerging from what they mean, as in a “historical ontology of ourselves” [27] or a “criticism of what we say, think, and do.” [28] From the epistemological perspective, subjectivism was considered to be a position to recognize, understand, and interpret the meaning of meanings and practices [28]. The information was analyzed using an interpretivist approach within a transverse timeframe, placing the C&R at the center of the phenomenon as the ones who construct, interpret, and modify reality [29, 30].

### Population and sample

Population. C&R who have direct contact with children diagnosed with some type of autoimmune disease. To determine the population, several phone calls and visits to specialists were made in order to come to agreements with them on when to apply the instrument within a 3-month period. Overall, 30 C&R agreed to participate in the study due to availability of time and the study’s relevance to their specialties and experience with children in clinical care or biomedical research. Inclusion criteria included clinicians of different specialties practicing in the city of Barranquilla, Atlántico/Colombia, who consented to participate in the study. The study also included those who had participated in research, have published scientific articles, or are or have been conducting research with minors. The research was approved by the research ethics committee of the doctoral program in bioethics at Universidad El Bosque, Bogotá / Colombia. The committee determined that the investigation is without risk according to resolution number 8430 of 1993 - Ministry of Health of Colombia. The Declaration of Helsinki was taken in its principle to safeguard the confidentiality of the personal information of the doctors and researchers who participated in the research.

Of the 30 interviewees, only 21 specialists met the inclusion criteria, including providing informed consent, providing clear responses to questions in both oral and written interviews, and having proven experience in clinical care or research with minors. Researchers who did not have a minimum of 4 years of clinical experience dealing with minors, decided not to participate in the study due to lack of time, or did not demonstrate a true disposition when answering the questions were excluded. A pilot test was conducted for internal validation of the instrument, which made it possible to improve the final drafting process of the questions to avoid preconceived ideas, as these could distort analysis of the information. It also allowed for closer adherence to ethical requirements, such as confidentiality through information encoding and interview application time. The instrument was also validated according to the criteria of experts from several universities who are knowledgeable on the subject. The final instrument had 15 open (10) and closed (5) questions, each having the purpose of measuring each of the categories that had been pre-established by the literature analysis.

The C&R had the following characteristics: 61.9% were aged 21–30 years, 23.8% were aged 31–40 years, and 9.5% were aged 60–69 years. The clinicians had an average experience of 8–9 years of clinical practice. One clinician who is also a researcher had 45 years of experience in both clinical and biomedical research. Finally, 66.7% of clinicians were female pediatricians and 14.3% were male. A gynecologist who stated having experience with girls with autoimmune diseases was interviewed, representing 4.8% of the population. A pediatrician and an infectologist (9.6%) also mentioned having experience in research with children. A rheumatologist/immunologist with experience treating children with autoimmune diseases who was also a nationally and internationally recognized researcher in polyarticular idiopathic arthritis represented 4.8% of the population. Of the interviewees, 9.5% stated having significant experience in research with children and adolescents (Table 1).

**Table 1:** Characteristics of the interviewees

Age (years)	n (%)		Clinical Specialties					Years of experience		Active projects with minors		Total
21–30	13 (62)	Gender	Gynecology	Pediatrics	Pediatrics and Infectious Diseases	Rheumatology and Immunology	Total	Average	8.8	No n (%)	Yes n (%)	n (%)
31–40	5 (24)		n (%)	n (%)	n (%)	n (%)		Median	4	19 (90.5)	0 (0.00)	21 (100)
50–59	1 (5)	Female	1 (4,8)	14 (66.7)	0 (0.00)	0 (0.00)	15 (71.4)	Mode	2			
60–69	2 (9)	Male	0 (0,00)	3 (14.3)	2 (9.6)	1 (4.8)	6 (28.6)	Minimum	1	0 (0.00)	2 (9.5)	
Total	21 (100)	Total	1 (4,8)	17 (81)	2 (9.6)	1 (4.8)	21 (100)	Maximum	45			

n: frequency, (%): percentage

The C&R included a [convenience sample] of rheumatologists, pediatricians, and pulmonologists, whose clinical practice has led to significant experience in caring for minors..

The data collection technique used was the semi-structured [focused] interview in Spanish, which was conducted within an average time of 15 to 20 min verbally and 20 to 30 min for those who preferred to write it.

The instrument was applied at the clinicians’ offices and in some cases at their residences according to the consideration of the subject researcher, thereby always ensuring a private environment to provide a strong sense of respect during participation and in the manner their responses were expressed. The setting was also conducive for clarification of elements at the time of the preliminary analysis of the information or for the investigator to clarify any aspect that could be misunderstood. This only occurred once with a single subject researcher minutes after completing the interview, and they were allowed to clarify their answer.

The interview went through an internal validation process that allowed us to propose alternative questions as long as they fulfilled the objective of the question. These methodological elements allowed for an analysis of the content of the meanings of autonomy, respect, and justice. This was accomplished through accounts of subjective experiences with objective analysis to understand the way in which physician–patient relationship-type power practices were constituted and the presence of the principles of bioethics: justice, non-maleficence, beneficence, and autonomy. Each of the respondents received a copy of the study at the end of the analysis and did not provide any comments regarding changes or completeness of information.

For qualitative data analysis, a description of the meanings of autonomy and influential practices by researchers and clinicians was established regarding a child’s autonomy according to their moral maturity. A semi-structured non-directive interview was conducted for this purpose, which was processed by analyzing the information to conceptualize the categories [31]. For these analysis dynamics, the Dragon Notes® and Atlas Ti 7.0® programs were used, for which the following analysis technique was proposed:

- Count: The way of finding, in a preliminary way, what is known and the emergence of the phenomenon regarding the meanings of autonomy and their relationships with influential practices.
- Identification of patterns and themes: the most frequently repeated words were tracked to identify categorical relationships and establish influential relationships with the child.
- Examination of the plausibility of the findings: the purpose was to contribute to the conclusions of the initial impressions, which made it possible to determine the guidelines in the analysis stages and contrast the construction of conclusions.

## Results

### Power relations: category analysis

The following categories were based on the influential relationships between C&R when considering the child as an autonomous subject.

Two types of influential relationships were mentioned in the interviewees’ discourse: the deliberative model, in which the C&R act as a mentor, contributing to the construction of the child’s autonomy and moral development, and the paternalistic model, in which the C&R act more as guardians and may even involve subjugation practices; i.e., not taking the child’s opinion into consideration and invoking the principle of beneficence as an ethical way to justify their practice based on regulations (principle of justice).

### Category: deliberative model

In the deliberative model, the C&R act as teachers and contribute to the child’s moral development under perceived elements such as autonomy and his or her understanding of the disease.

In their role as mentors, the C&R accompany the child and his or her family actively and reflectively through a doctor–child–guardian-type relationship, which favors the child’s state of mind. This also contributes to motivating the child to pursue treatment and to understand the consequences of not undergoing it. However, the C&R understand the importance of the beliefs of the child’s family, the configurative elements of cognition typical of the child’s age, and their experience with illness. In other words, the C&R favor constructing the child’s moral judgments to establish a healthy relationship with themselves and their environment, thereby strengthening their will for better care as well as their sense of utility, as shown in the following statements in the constant dialogue category:

### Emerging category: constant dialogue

C&R mention the need to promote dialogue with the child to establish empathic links using different methods to achieve their understanding and increase their responsibility and autonomy.

Question: Describe how you would tell a child about a new treatment and what determines the degree of information provided.

- *Coded speech [E-21–1:41]: First, ease of communication to provide clear information and more so in adolescents because they have difficulty in accepting the disease. Therefore, we must begin to explain the disease to the child in a basic way because information reassures them and more so if it is repetitive and time is taken to provide the information.*
- *Coded speech [E-10–1:42]: I would explain the benefits and possible adverse side effects verbally using brochures with text and images to achieve greater understanding.*

### Emerging category: participation

According to the C&R, structuring the child to strengthen their autonomy and moral development implies gaining their participation as an active moral subject and holding them accountable for their self-care practices in terms of following the treatment instructions, i.e., being responsible for their actions.

Question: From your clinical or investigative practice, how do you think you contribute to the strengthening of moral development, which is understood to be the child’s independence, freedom, and responsibility to make moral judgments?

- *Coded speech [E-7–1:209]: In clinical practice, it is the contact with the patient when addressing adverse situations by means of a good doctor–patient relationship that leads to better results and also to the patient’s cognitive development after the event.*
- *Coded speech [E-9]-[1:212]: Contributing to their ability to listen and their responsibility for making decisions regarding their health.*
- *Coded speech [E-14]-[1:216]: Making them participate in their diagnosis, prognosis, and treatment.*
- *Coded speech [E-19]-[1:220]: Contributing to their autonomy so that they participate directly in managing their treatment.*

### Emerging category: knowledge

For C&R, the child’s knowledge of their illness contributes to the type of doctor–patient relationship that may emerge, especially the deliberative one, which directly influences the child’s will, as in the way that they obtain and analyse information obtained on the internet, and their mood and motivation, as stated in the following statements:

Question: Based on your experience, what would a child’s ability to make an important decision regarding their health, specifically their self-care, depend on?

- *Coded speech [E-6]-[1:131]: On their age, on their knowledge of their illness, on the security that their environment gives them, and on their experience with the disease.*
- *Coded speech [E-6]-[1:133]: It depends on their degree of knowledge of their disease and the commitment it generates, recognizing that given its chronicity, they should practice better self-care to counteract relapses.*

Question: Do you consider that the experience of pediatric patients with respect to their autoimmune disease contributes to the development of their moral judgments? If your answer is yes, justify your answer.

- *Coded speech [E-5]-[1:155]: Currently, the availability of information and technological advances make these type of patients more informed about their disease and allows them to develop moral judgments at a very young age.*
- *Coded speech [E-21]-[1:166]: Yes, as long as they are motivated to understand and practice their care in the best possible way with or without their parents’ support.*

Question: Doctor, from your point of view, can maturity that may be demonstrated by the child regarding self-care be extrapolated to other areas of their life and, if so, in what sense?

- *Coded speech [E-18]-[1:184]: Yes, because of the positive stance they assign to their self-care projects them to other areas or aspects of their life.*

### Category: paternalistic model

C&R state that they contributed to the child’s development or moral maturity based on elements such as acceptance. When the C&R determine that the child does not have a good understanding of the information and their common sense is not developed, they consider that it is owing to a lack of cognitive capacity, which may be affected by the type of illness that afflicts them, the lack of maturity at their age, and suffering when experiencing pain or fear. All these factors prevent the child from reaching an adequate degree of responsibility for their self-care. Therefore, the clinician has to behave as a guardian owing to the child’s high vulnerability and even refer them to psychological support along with the parents. Thus, C&R are in a doctor–guardian–child-type relationship, as represented in the following statements:

Question: Doctor, how do you consider a child with autoimmune disease?

- *Coded speech [E-6]-[1:257]***:** *A subject who must receive special care and assistance because of their vital vulnerability.*
- *Coded speech [E-18]-[1:267]***:** *A subject of care with diminished autonomy.*

### Emerging category: submission

This emerging category is related to the fact that C&R immediately ignore the child’s autonomy when the child does not accept what is set out by the C&R or presents arguments that go against the treatment proposed by the clinician.

Question: What elements do you think are important when considering whether a child can make a decision regarding the new treatment?

- *Coded speech [E-1]-[1:91]: An autonomous child, that is, with recognition of their responsibility, because their disagreement denotes their lack of maturity.*
- *Coded speech [E-9]-[1:102]: Accepting the benefits that the product gives them.*

### Emerging category: acceptance

C&R consider that a child should accept the treatment or research protocol designed for their care without further conflict as a way of proving their maturity, as represented in the following statements:

Question: What elements are important to consider when deciding whether or not a child can make a decision regarding the new treatment?

- *Coded speech [E-9]-[1:102]: Accepting the benefits the product gives them.*

C&R also regard the effects of medication or treatment in general, as stated in the following responses:

- *Coded speech [E-6]-[1:114]: They should accept the adverse effects.*

### Emerging category: Guardian

C&R consider that a child’s vulnerability is owing to elements of their age or pathological status and the characteristics of the medications. Therefore, it is the clinician’s duty to be guided by elements of his or her judgment as an expert, beyond what the child or even the parents may consider. Therefore, clinicians, above all, consider themselves as guardians, bearing in mind the principles of bioethics (justice and non-maleficence) of childcare, as represented in the following statements:

Question**:** Based on your experience, what would the child’s ability to make an important decision regarding their health, specifically their self-care, depend on?

- *Coded speech [E-14]-[1:143]: The limitations or adverse effects that may occur due to medication administration.*

Question**:** Do you consider that the experience of pediatric patients with some type of autoimmune disease contributes to the development of moral judgments? If your answer is yes, justify your answer.

- *Coded speech [E-12]-[1:161]: Yes, they are patients with few options who require the implementation of new support means.*

## Discussion

What autonomy means, as stated by C&R with respect to the child, is what regulates the influential practices when obtaining informed consent to consider the child as an active moral subject. However, we must consider that factors inherent in the child as well as external factors (family, clinical, research, and sociocultural factors) contribute to the formulation and implementation of strategies that strengthen the child’s moral development; this element has been raised as a process of Differentiated Objectivization [Educative] (POED) [32, 33].

POED should give the child a sense of usefulness so that they recognize their responsibilities in terms of their care, and therefore, their moral capacity to help them make the best decision based on heteronomy, thereby supporting the child’s capacity for action (agency) [15]. In turn, the principles of bioethics, such as a respect for others, beneficence, and justice, guide the ethical reflections of C&R when considering the child at a given time as an autonomous subject to give consent, keeping in mind the idea of the child’s active conation, as explained by Nussbaum [15], wherein the practices of C&R must go beyond that stipulated by the norm when considering the child as an adult based on purely regulatory arguments, for example, age, which may not be related to their degree of maturity when they face their self-care and protection, possibly owing to the child’s experience with the disease.

Foucault stated that the idea that the child’s moral recognized by C&R must be based on a POED is confirmed, i.e., through the influential relationships that emerge while obtaining informed consent [34]. To provide the POED, the characteristics of each minor and degree of maturity that emerges from their self-care practices must be taken into account as well as their understanding of the risks and benefits of the treatments. The above requires practices of subject objectification that will depend on internal and external elements, i.e., ‘the subject is found to be divided within or divided from others’, wherein this process is ‘objective’ [34].

In short, the child’s vulnerability should not justify a violation of their right to consent. Conversely, it is the moral duty of C&R to contribute to the development of their autonomy based on the informed consent process. According to Herder, human beings come into the world “weak [ …], needy [ …], lacking the teachings of nature [ …], lacking skills and talents,” as quoted in Kottow (2012) [35]. However, “man is nothing more than a blade of grass, the most fragile thing in nature, but it is a blade of grass that thinks.” [36].

According to the above, considering a child as mature depends a lot on the judgment and common sense developed as a result of their experience with the disease. Authors such as Nunner-Winkler and Sodian consider that a child’s maturity is related to their experience with the disease when recognizing risks and benefits in line with their negative or positive emotions, which structures the way of being and existing in the world that could be perceived by the clinician as autonomy [37], which was proved in this study.

In summary, the legal framework that defines the child’s autonomy and experiences defines their moral development. This statement arises owing to their first being the legal obligation to take the child’s consent and the legal guardian’s consent and secondly, from the moral duty of the C&R to ensure the child’s moral development by strengthening their self-care based on the understanding and experience with the disease that afflicts them.

Regarding the influential practices in the doctor–patient relationship, it was found that the principles of autonomy and beneficence emerged in the deliberative model, turning the relationship into a trinomial of active participation (C&R–child–guardian). The principle of beneficence was found to be at the time that the C&R provide elements from the communication process related to providing clear information, which contributes to the child’s moral development so that he or she is aware of their decision and of the different risks and benefits to which they are exposed if they do not think positively about their treatment or their duty toward future generations when talking about biomedical research. This means that C&R increasingly require communication skills but also require knowledge and ethical attitudes to assess a child’s competencies, Drane (1999) designed a methodology in which the criterion or principle of proportionality is taken into account for clinical decision making, considering the severity of the decision and the degree of moral competence of the child [37].

Michaud mentions that there is a need to apply the principles of bioethics, but the rights of the child should also be considered, and they should be considered as subjects with their own personality and having rights themselves [38]. In this sense, should a child be considered mature or autonomous? C&R affirm that this acknowledgement depends a lot on their level of understanding of the disease and how they practice self-care in addition to the child’s degree of dependence on their parents.

According to Labouvie-Vief, there is a relationship between consent and parental authorization, wherein each has a different purpose [39]; for example, the guardian’s supervision is intended to protect the child from taking unreasonable risks and to strengthen their autonomy when making fair and responsible decisions for their health. Conversely, consent is a communicative process with which one must try and strengthen the sense of usefulness and ensure that the child adheres to treatment for life. Even 9-year-old children have demonstrated the ability to make reasonable choices as long as these choices are explained to them using appropriate language [40, 41].

## Conclusions

It is evident that a child’s moral structuring depends on individual characteristics particular to their evolutionary stage and on their degree of understanding of the facts that surround them as well as their experience with the disease. Thus, they initiate the differentiation suggested in POED and recognize the risks and benefits of continuing with their self-care, which may be above their subjective wishes [inclinations] and put their life in danger. Therefore, C&R also consider the active participation of parents or legal guardian to be important. However, even if it seems paradoxical, when considering the child as an active moral subject, they must respond to different degrees of independence from their family.

Conversely, submission of the child is presented as an influential practice of extreme paternalism when the C&R perceives a lack of desire for their care in the child or that their understanding is below the expected levels; therefore, the guardian’s direct intervention is also necessary. This does not happen when it comes to research because the guardian is informed about the care and if it is accepted, the minor becomes a passive subject in which consent could take the form of a simple step required by a regulatory process. Although in research, the guardian is addressed, and if they do not accept it, the child is not exposed to considering the information provided, even vice versa, and if the child does not accept it, it is advisable not to include them in the study.

In POED, different nuances could be expected regarding the practices based on the different models of the doctor–patient relationship. According to the deliberative model, C&R hope that the child is aware of their illness and acquire moral awareness of their care to the extent that their experiences have a positive impact on their health. This element of moral development will depend on the child’s age and on the degree of independence they may have from their family and the level of understanding of the information provided, that is, their common sense. The opposite is the paternalistic model, in which submission is a way of structuring the child because they are considered to be a passive subject of extreme care, worthy of protection and care. Therefore, C&R consider that structuring a child and perceiving them as an autonomous subject is only possible when they adhere to treatment and responsible self-care, regardless of their parents’ support, bearing in mind that it also depends on the type of disease.

### Limitations

Limitations were related to selective memory bias in the research subjects. In this sense, an attempt was made to control the bias by means of complementary questions, to avoid, for example, that they only recalled negative or positive events, tending to exaggerate or beautify the phenomenon, that is, the positive or negative assent to participate in research or treatment.

We emphasize that the data cannot be generalized to all populations and sociocultural contexts, since the conclusions may vary due to the perception that clinicians and researchers have on the moral structure of the minor, which can be observed during the process of obtaining consent informed.

## Data Availability

The data sets generated and / or analyzed during the current study are not publicly available because we will conduct other analysis from which we expect future publications and also support other projects of students in doctoral training but are available from the corresponding author upon reasonable request.

## Abbreviations

POED: *Differentiated Objectivization Process [Educative]*
C&R: Clinicians and Researchers
Child: Child and adolescents
